# Special Issue: Ribozymes and RNA Catalysis

**DOI:** 10.3390/molecules22050789

**Published:** 2017-05-11

**Authors:** Sabine Müller

**Affiliations:** Institut für Biochemie, Ernst-Moritz-Arndt-Universität Greifswald, Felix-Hausdorff-Str. 4, Greifswald 17489, Germany; smueller@uni-greifswald.de

Over the past 35 years, RNA has become a molecule of utmost interest for researchers in the life sciences. The many functions that RNA fulfills in the cellular machinery have been elucidated with constant progress, revealing a complex network of RNA-mediated regulation of key processes in the cellular life cycle. First examples of RNA catalysis were discovered in the early 1980s, and it was nothing less than the Nobel prize that was awarded for this discovery [1,2]. Since then, a large number of RNA catalysts, found in nature or developed in the test tube, have accompanied these first examples, and the term “ribozyme” has found entry in text books and encyclopedias. This Special Issue has aimed to collect state-of-the-art research and review articles on ribozymes, the study of their structure and function as well as engineering of ribozymes into new applications.

Two review articles highlight important aspects of ribozyme structure and function. Lau and Ferré-D’Amaré discuss the functional plasticity of ribozyme folds in their review [3]. The function of ribozymes requires the formation of active sites decorated with RNA functional groups within defined three-dimensional (3D) structures. As follows from a number of recent results, RNA activity cannot be simply optimized by sequence changes. Instead, small changes in the genotype of an RNA that has reached a stable three-dimensional fold, can induce distinctly different biochemical activities. Lau and Ferré-D’Amaré discuss how this functional plasticity can affect the adaptation of organisms to changes in selective pressure and how it can be used for biotechnological application [3]. The review by Lee and Lee looks at the structure, biochemistry and catalytic mechanism of novel self-cleaving ribozymes [4]. For a long time, 10 classes of ribozymes existing among contemporary organisms were known, the hammerhead and hairpin ribozyme being probably the most studied and best characterized examples. The newly discovered twister, twister-sister, pistol and hatchet ribozyme (keeping with the tradition of choosing ribozyme names in relation to their secondary structure) now add up to 14 ribozyme classes in total. As mentioned above, the hammerhead ribozyme belongs to the most prominent examples of naturally occurring ribozymes. It was the first of the small self-cleaving ribozymes that was discovered, and over the years it has been extensively studied. Still, the hammerhead ribozyme has not yet revealed all its secrets. It is widespread in nature and appears in rather diverse genetic contexts. However, the biological role and precise function of hammerhead ribozyme motifs in the genomes of organisms in all kingdoms of life are not yet well understood. In this Special Issue, De la Pena et al. review the long history of the small ribozyme [5].

In addition to the ongoing research into the structure and mechanism of ribozymes, RNA catalysis has also inspired a large number of efforts into RNA engineering and design. This is the topic of the review of Müller on trans-splicing group I Intron ribozymes [6]. It summarizes the different variants of trans-splicing group I Intron ribozymes that have been generated in the Müller laboratory, and highlights their potential as tools in therapy as well as their relevance as model systems for RNA evolution in cells. Group I introns are also the focus of two research papers in this Special Issue [7,8]. Andersen et al. report on the processing of group I introns in the myxomycete *Didymium iridis* to form full-length circular RNAs and highlight the structural aspects explaining their relative stability [7].

The discovery of ribozymes has revitalized the RNA world theory, and ever since this discovery, much effort has gone into the development of ribozymes with useful activities in a scenario of life based on RNA functioning as both a genome and genome-encoded catalyst. Along this line, the paper by Agmon follows the interesting hypothesis that a proto-ribosome, comprised of a dimer of tRNA-like molecules, is still embedded in the contemporary ribosome and provides a computational analysis of crystal structures to confirm it [9]. Satterwhite et al. delve into the RNA world reporting on the mechanistic details of group I intron ribozymes that help synthesize other ribozyme genotypes by recombination in an autocatalytic fashion [8]. Apart from recombination, self-replication is another important issue in RNA world scenarios. Olea and Joyce report on a RNA Ligase that undergoes exponential amplification, optionally also in a ligand-dependent manner [10]. Nevertheless, the key issue of the actual study is the development of an assay for real-time detection of this autocatalytic self-replication, and the demonstration of the potential of ligand-dependent self-replication for diagnostics and biosensing.

Overall, research into ribozymes and RNA catalysis has remained a very exciting field with unchanged potential for new discoveries. This Special Issue illuminates just a small window in this broad field. Ribozyme research has not lost the intriguing and highly motivating flair of the early days, when the first reports of ribozymes occurring in nature appeared. There is still much to be learned: from the discovery of novel catalytic RNA motifs in nature and novel genetic locations of known ribozymes, over the elucidation of ribozyme structure and its catalytic mechanism, up to engineering and design towards application in diverse fields. Ribozymes and RNA catalysis are of ongoing interest to the research community.
